# Effect of corticosteroid injection for trochanter pain syndrome: design of a randomised clinical trial in general practice

**DOI:** 10.1186/1471-2474-8-95

**Published:** 2007-09-19

**Authors:** Aaltien Brinks, Rogier M van Rijn, Arthur M Bohnen, Gabriël LJ Slee, Jan AN Verhaar, Bart W Koes, Sita MA Bierma-Zeinstra

**Affiliations:** 1 Department of General Practice, Erasmus MC, University Medical Centre Rotterdam, the Netherlands; 2 Department of Orthopaedics, Erasmus MC, University Medical Centre Rotterdam, the Netherlands

## Abstract

**Background:**

Regional pain in the hip in adults is a common cause of a general practitioner visit. A considerable part of patients suffer from (greater) trochanteric pain syndrome or trochanteric bursitis. Local corticosteroid injections is one of the treatment options. Although clear evidence is lacking, small observational studies suggest that this treatment is effective in the short-term follow-up. So far, there are no randomised controlled trials available evaluating the efficacy of injection therapy.

This study will investigate the efficacy of local corticosteroid injections in the trochanter syndrome in the general practice, using a randomised controlled trial design. The cost effectiveness of the corticosteroid injection therapy will also be assessed. Secondly, the role of co-morbidity in relation to the efficacy of local corticosteroid injections will be investigated.

**Methods/Design:**

This study is a pragmatic, open label randomised trial.

A total of 150 patients (age 18–80 years) visiting the general practitioner with complaints suggestive of trochanteric pain syndrome will be allocated to receive local corticosteroid injections or to receive usual care. Usual care consists of analgesics as needed. The randomisation is stratified for yes or no co-morbidity of low back pain, osteoarthritis of the hip, or both. The treatment will be evaluated by means of questionnaires at several time points within one year, with the 3 month and 1 year evaluation of pain and recovery as primary outcome. Analyses of primary and secondary outcomes will be made according to the intention-to-treat principle. Direct and indirect costs will be assessed by questionnaires. The cost effectiveness will be estimated using the following ratio: CE ratio = (cost of injection therapy minus cost of usual care)/(effect of injection therapy minus effect of usual care).

**Discussion:**

This study design is appropriate to estimate effectiveness and cost-effectiveness of the injection therapy. We choose to use a pragmatic study design and are thus not able to study specific effects of the injection with corticosteroids. A distinction between placebo effect of the injection and specific effects of the corticosteroids is therefore not possible.

**Trial Registration:**

The trial is listed in the Dutch Trial Registry with the number ISRCTN16994576

## Background

One of the more common pain syndromes of the in the hip in adults is known as trochanteric pain syndrome (TPS) or trochanteric bursitis. It is considered to have the following characteristics: chronic, intermittent pain at the lateral site of the upper limb, sometimes radiating to the lateral aspect of the hip or lateral thigh and increasing at physical activity [1-4]. Lying on the affected site increases the pain and can thereby disturb sleep and/or rest. At physical examination palpation of the greater trochanter is painful [1-4].

The prevalence is higher among females than among males (rate 4:1) and the incidence is highest between the ages 40 to 60 years [4,5]. A recent prospective study in a Dutch general practice population showed an incidence of 5.6 patients per 1000 adults in one year (unpublished pilot data). In a retrospective study, Lievense et al. found an incidence of 1.8 per 1000 in one year [5].

Earlier, the same clinical manifestations were known as trochanteric bursitis, although clinical manifestations of inflammation almost never occur [1,2,4]. Tendinitis of the insertion of the m. gluteus medius is suggested as another cause of pain at this site. It might also be a combination of bursitis and tendinitis. Because the exact aetiology is not known, Collee et al. suggested in 1991 to classify the clinical manifestations as "greater trochanter syndrome" [6].

Observational studies showed that in most of the cases with local pain at this site co-morbidity exists. About two-thirds of the patients with TPS have also low back pain or osteoarthritis of the hip [1,3,6,7].

Many GPs inject corticosteroids combined with an anaesthetic agent at the most painful site with the expectation that the pain will decrease. There is no conclusive evidence that these injections are effective, although small observational studies suggest that injections with corticosteroids are effective in the short-term follow-up [2,5,7,8]. No randomised controlled trials are available evaluating the benefit of injection therapy for this disorder. Other common treatment options are pain relief with analgesics, physiotherapy, a surgical release of the iliotibial tract, removal of the bursa, or a trochanteric reduction osteotomy [9].

This study we will investigate the efficacy and cost effectiveness of local corticosteroid injections in TPS, using a randomised controlled trial design. We also investigate the role of co-morbidity in relation to the efficacy of this local corticosteroid injection.

## Methods/Design

### Study design

This study is a pragmatic, open label randomised trial.

The local Medical Ethics Committee of the Erasmus University Medical Center, Erasmus MC, has approved the trial. The trial is included in the Dutch Trial register (ISRCTN 16994576).

All patients will give written informed consent.

### Patient selection

GPs participating in the HONEUR research network of the Erasmus MC and other interested GPs in the area will be invited to participate in the study. The HONEUR GP research network consists of 40 GP practices which are connected to the Department of General Practice in order to participate in regular research projects. They will be asked to select patients aged 18 to 80 years visiting the GP with the following symptoms: pain persisting for more than one week in the lateral region of the hip or thigh with tenderness at palpation of the greater trochanter with one of the two following characteristics [1-4,10]:

1). Severe pain at palpation of the greater trochanter, but uncertainty as to whether the patient recognizes the pain as that for which he or she visits the GP.

2). Local tenderness when the area of the great trochanter is palpated and the patient recognizes the pain as that for which he or she visits the GP.

Excluded are patients who are unable to understand the Dutch questionnaires, patients who have consulted the GP with the same complaints in the previous year and had any intervention, or are operated on in the same region, or have systemic neurological or rheumatologic disorders.

### Procedures

Patients who are eligible for the study and show interest to participate will receive written study information from their GP, as well as the baseline questionnaire and the informed consent form. If they show interest the GP will fax their contact data to the researcher together with the findings of physical examination on a standardized form. One of the investigators will contact the patient to ask if they have any additional questions and will assess the suitability to participate in the study. If the patient still wants to participate and is eligible, we ask them to return the baseline questionnaire and informed consent form.

When we receive the baseline questionnaire, the patient will be classified as having comorbidity or not. If the question: "Do you suffer from low back pain" is answered positively as often or continuous, we classify the patient as having low back pain comorbidity. The ACR criteria for osteoarthritis of the hip, using history and physical examination (painful or decreased internal rotation and flexion of the hip as performed by the GP) are used to decide whether the patient has osteoarthritis of the hip as comorbidity [11].

### Randomisation

After receiving the baseline questionnaire and informed consent, an independent person will randomise each patient based on computerized randomisation lists to either receive the injection therapy or the usual care, consisting of analgesics as needed. This randomisation is stratified for yes or no comorbidity and uses randomisation block sizes of ten, yielding four strata: one lacking comorbidity, one with low back pain, one with osteoarthritis, and one stratum with both.

### Intervention

The GP and the patient will be informed about the treatment that the patient will be given as soon as randomisation has taken place. The GPs participating in the study have been trained by us to give the injection according to a standard procedure: i.e. to use 40 mg triamcenolon acetate combined with lidocaine 1 or 2% in a 5 ml syringe. They are trained to mark the most painful point with a pen or pencil and desinfect the site. The needle is inserted perpendicular to the skin and directed down to the point of maximal tenderness, 1 ml of the substance will be injected at that point, then the needle should be moved to another place in the painful area and the same procedure should be repeated until the syringe is empty. In case of lack of efficacy, or only temporary effect, this procedure may be repeated after a period of 3 weeks up to 3 months

After the injection the GP will fax us a form with details of the injection given, e.g. the volume that is injected, if the injection was painful, or if there was pain relief after injection or direct side effects.

The control group will receive usual care consisting of analgesics as needed; all patients are free to receive additional treatment from a physiotherapist, although this is not advocated by the investigators.

### Questionnaires

The primary outcome measurements will be experienced recovery at 3 months and at 1 year, measured on a 7-point Likert scale (1 = fully recovered till, 7 = worse than ever) and severity of pain during the last week measured with a numeric rating scale (0 = no pain, 10 = worst conceivable pain). Medical consumption of the patient (e.g. medication, visits to the GP or physiotherapist, hospital treatment and diagnostic tests) will be measured as direct cost. The PRODISC questionnaire will be used to measure the cost effectiveness and will measure the indirect costs, expressed in work staff absence and loss of productivity in paid work, or loss of productivity in not-paid work [12]. The WOMAC (Western Ontario and McMaster University Osteoarthritis Index) is recommended by the Osteoarthritis Research Society for use in clinical trials in people with hip osteoarthritis to measure pain and disabilities [13]. The HOOS (hip disability and osteoarthritis outcome score) [14] is developed as an extended version of the WOMAC, to evaluate the whole domain of patient-relevant outcome in young and active patients and is recently validated in the Dutch language [15]. The HOOS consists of 5 subscales; Pain, other Symptoms, Function in daily living (ADL), Function in sport and recreation (Sport/Rec) and hip-related Quality of life (QOL). Both the WOMAC and HOOS will be used in this study. We use the EuroQol (EQ5D) as instrument to measure quality of life [16].

Finally, we ask the patients who received an injection, to report any side effects of the injection. Table 1 shows the timing of measurements.

**Table 1:** Timing of study questionnaires

	baseline	6 weeks	3 months	6 months	9 months	12 months
Severity of pain: VAS (1–10)	x	x	x	x	x	x
Experienced recovery: Likert (1–7)		x	x	x	x	x
HOOS including WOMAC	x	x	x	x	x	x
Prodisq + medical consumption	x		x	x	x	x
EQ 5 D	x		x	x	x	x
Side effects of injection		x	x	x	x	

### Sample size

The sample size was calculated to detect an increase in recovery rate of 25% in the intervention group after three months of follow-up (45% recovery in the control group versus 70% recovery in the intervention group). With power 0.8 and alpha 0.05 (two-sided tested) and with a dropout rate of 10%, a total number of 75 patients in each group are needed.

### Data analyses

Difference between the groups in the primary outcome will be analysed based on the basis of the "intention-to-treat" principle. Differences in continuous outcome measures between the groups will be analysed with linear regression techniques and differences in dichotomous outcomes (recovery dichotomised to totally recovered or almost totally recovered versus slightly recovered and less) will be analysed with logistic regression techniques. Baseline differences between the groups will be assessed and checked whether these influence the outcome of the study. Baseline variables that change the outcome by 10% or more will be regarded as confounders and will therefore be added to the regression models.

Regression models will also be used to study effect modification of comorbidity. A cost-effectiveness analyses will be performed from a social and a patient perspective, looking at differences in direct and indirect health care cost between the two groups.

If the trial does not show a difference in disease parameters (VAS and WOMAC) and quality of life (EuroQol) between the groups, the analysis will be reduced to a cost minimisation analysis. This form of analysis evaluates the efficacy of treatment based solely on direct and indirect costs. If the study does find a positive difference in disease parameters and/or quality of life in the injection group, a cost-effectiveness ratio can be determined.

## Discussion and current status

In this study design we compare two types of therapy which are frequently used in general practice. This study design is appropriate to estimate effectiveness and cost-effectiveness of the injection therapy. We choose to use a pragmatic study design and are thus not able to study specific effects of the injection with corticosteroids. A distinction between placebo effect of the injection and specific effects of the corticosteroids is therefore not possible.

The study is executed in general practice and may therefore not apply to patients in secundary care.

No disease specific questionnaires are available for this disorder. Therefore generic outcome measures (recovery and pain severity) were chosen as primary outcome measures. However, region specific questionnaires for osteoarthritis were included as secondary outcome measures. These questionnaires allow us to analyse subscales like for instance the WOMAC pain and the WOMAC function; subsequently osteoarthritis specific questions on stiffness (WOMAC stiffness) can validly be omitted.

Patients included in our trial have symptoms of TPS solely, or have these symptoms in co-occurrence with low-back pain or osteoarthritis of the hip. To include patients with such co-morbidity maybe questionable because the TPS may be due to such morbidity. Therefore injection therapy for TPS in such morbidity possibly shows different effectiveness. We chose to include these patients because in common practice these patient also receive injection therapy. We, however, used a design in which we stratify for co-morbidity or not.

The current status of the study is that of a total of the 80 GPs participating in the study. However, until now only 43 GPs included 90 patients. The total study population is expected to be recruited by February 2008. The first short-term results (3 months of folluw-up) will be available at mid-2008.

## Competing interests

ZonMw, the Netherlands Organisation for Health Research and Development supported this study.

The authors have no competing interests.

## Authors' contributions

SMABZ, AMB, JANV, BWK and GLJS participated in the design and coordination of the study. AB and RMR coordinate the trial and are responsible for data acquisition. AB and SMABZ prepared the article.

All authors have read and approved the final article.

## Pre-publication history

The pre-publication history for this paper can be accessed here:


